# Supplementary material for: Homeostatic plasticity fails at the intersection of autism-gene mutations and a novel class of common genetic modifiers
Source: eLife. 2020 Jul 1;9:e55775. doi: 10.7554/eLife.55775 (PMC7394548; doi:10.7554/eLife.55775)
Supplement: Supplementary file 1. — Each table is referred to independently in the text. [file elife-55775-supp1.docx]

**Table S1 (referring to Figure 1)**

Human ASD genes, associated disorders and links to Web-based documentation.

| **Gene name** | **Associated disorders** | **SFARI link** |
| --- | --- | --- |
| RIMS1* | ASD | [https://gene.sfari.org/database/human-gene/RIMS1](https://gene.sfari.org/database/human-gene/RIMS1) |
| WDFY3 | ADHD, ASD, DD/NDD, EPS | [https://gene.sfari.org/database/human-gene/WDFY3](https://gene.sfari.org/database/human-gene/WDFY3) |
| CHD2 | ASD, ID, EPS, ADHD, EP, DD/NDD | [https://gene.sfari.org/database/human-gene/CHD2](https://gene.sfari.org/database/human-gene/CHD2) |
| CHD8 | ASD, ID, DD/NDD, EPS | [https://gene.sfari.org/database/human-gene/CHD8](https://gene.sfari.org/database/human-gene/CHD8) |
| ASH1L | ASD, ID, DD/NDD, EPS | [https://gene.sfari.org/database/human-gene/ASH1L](https://gene.sfari.org/database/human-gene/ASH1L) |

*Mutations in RIMS1 have been identified as the cause of cone-rod dystrophy type 7 (CORD7) [OMIM:603649]

**Abbreviations: **ASD**: Autism Spectrum Disorders. **ADHD**: Attention Deficit with Hyperactivity Disorder. **ID**: Intellectual Disability. **EPS**: Extrapyramidal Symptoms. **DD/NDD**: Developmental Delay/Neurodevelopmental Disorder. **EP**: Epilepsy.

**Table S2 (referring to Figure 2)**

Average values obtained during rapid-throughput forward genetic screen.

| **Deficiency ID** | **Quantal content** | **mEPSP** | **n** |
| --- | --- | --- | --- |
| wild type | 77.10 | 0.44 | 36 |
| *rim*^103^/+ | 64.36 | 0.40 | 30 |
| 7562 | 27.37 | 0.43 | 4 |
| 7564 | 48.27 | 0.40 | 4 |
| 7565 | 56.02 | 0.57 | 3 |
| 7567 | 40.35 | 0.67 | 4 |
| 7571 | 43.70 | 0.50 | 4 |
| 7572 | 54.49 | 0.41 | 3 |
| 7574 | 44.44 | 0.41 | 3 |
| 7576 | 54.60 | 0.32 | 3 |
| 7577 | 53.89 | 0.34 | 4 |
| 7578 | 30.80 | 0.50 | 7 |
| 7580 | 34.69 | 0.49 | 4 |
| 7584 | 44.61 | 0.42 | 4 |
| 7585 | 48.54 | 0.52 | 4 |
| 7586 | 50.32 | 0.39 | 4 |
| 7588 | 45.77 | 0.40 | 4 |
| 7589 | 42.64 | 0.47 | 4 |
| 7591 | 56.56 | 0.47 | 3 |
| 7594 | 41.65 | 0.44 | 4 |
| 7595 | 45.54 | 0.40 | 4 |
| 7596 | 16.21 | 0.35 | 4 |
| 7600 | 40.11 | 0.51 | 4 |
| 7601 | 48.09 | 0.37 | 4 |
| 7602 | 65.69 | 0.28 | 4 |
| 7609 | 53.41 | 0.49 | 7 |
| 7610 | 55.96 | 0.38 | 4 |
| 7611 | 39.35 | 0.41 | 4 |
| 7612 | 38.44 | 0.41 | 10 |
| 7613 | 52.13 | 0.36 | 8 |
| 7614 | 58.94 | 0.36 | 6 |
| 7616 | 46.10 | 0.39 | 6 |
| 7619 | 38.46 | 0.45 | 3 |
| 7620 | 50.12 | 0.39 | 4 |
| 7621 | 35.71 | 0.39 | 3 |
| 7622 | 37.22 | 0.42 | 4 |
| 7624 | 39.20 | 0.50 | 4 |
| 7635 | 38.01 | 0.45 | 4 |
| 7637 | 79.70 | 0.35 | 3 |
| 7638 | 57.85 | 0.41 | 4 |
| 7644 | 42.52 | 0.41 | 4 |
| 7648 | 41.53 | 0.42 | 6 |
| 7649 | 65.52 | 0.38 | 3 |
| 7652 | 42.57 | 0.48 | 4 |
| 7653 | 45.10 | 0.38 | 3 |
| 7657 | 31.12 | 0.50 | 4 |
| 7658 | 38.60 | 0.43 | 4 |
| 7659 | 82.29 | 0.35 | 4 |
| 7660 | 37.36 | 0.46 | 3 |
| 7662 | 38.41 | 0.62 | 4 |
| 7664 | 56.74 | 0.31 | 4 |
| 7666 | 40.24 | 0.52 | 2 |
| 7672 | 76.23 | 0.41 | 3 |
| 7674 | 43.44 | 0.43 | 2 |
| 7675 | 45.67 | 0.41 | 3 |
| 7676 | 49.39 | 0.51 | 4 |
| 7680 | 63.25 | 0.41 | 2 |
| 7681 | 30.03 | 0.60 | 4 |
| 7682 | 53.21 | 0.41 | 5 |
| 7694 | 47.32 | 0.49 | 4 |
| 7731 | 47.91 | 0.48 | 4 |
| 7734 | 74.78 | 0.30 | 3 |
| 7737 | 49.26 | 0.56 | 2 |
| 7742 | 35.13 | 0.40 | 4 |
| 7743 | 61.90 | 0.42 | 4 |
| 7746 | 39.72 | 0.43 | 4 |
| 7921 | 36.18 | 0.38 | 3 |
| 7922 | 42.65 | 0.53 | 8 |
| 7927 | 45.27 | 0.37 | 4 |
| 7929 | 37.49 | 0.41 | 3 |
| 7930 | 72.49 | 0.36 | 2 |
| 7937 | 41.84 | 0.47 | 5 |
| 7938 | 51.26 | 0.43 | 3 |
| 7947 | 56.28 | 0.40 | 4 |
| 7951 | 75.80 | 0.31 | 4 |
| 7952 | 42.65 | 0.43 | 4 |
| 7953 | 35.49 | 0.38 | 4 |
| 7957 | 37.73 | 0.27 | 3 |
| 7958 | 36.40 | 0.34 | 4 |
| 7959 | 38.30 | 0.39 | 4 |
| 7961 | 63.59 | 0.40 | 4 |
| 7963 | 32.17 | 0.35 | 4 |
| 7975 | 66.10 | 0.37 | 4 |
| 7976 | 55.03 | 0.31 | 7 |
| 7977 | 48.99 | 0.42 | 3 |
| 7980 | 45.59 | 0.41 | 10 |
| 7981 | 55.23 | 0.41 | 3 |
| 7983 | 43.12 | 0.43 | 4 |
| 7990 | 33.10 | 0.38 | 4 |
| 7992 | 21.93 | 0.40 | 4 |
| 7994 | 60.64 | 0.33 | 4 |
| 7997 | 29.10 | 0.40 | 2 |
| 8057 | 36.61 | 0.37 | 4 |
| 8061 | 41.75 | 0.45 | 2 |
| 8063 | 46.99 | 0.41 | 2 |
| 8068 | 36.55 | 0.45 | 7 |
| 8070 | 44.21 | 0.42 | 4 |
| 8074 | 53.10 | 0.37 | 4 |
| 8083 | 42.69 | 0.36 | 10 |
| 8097 | 26.16 | 0.40 | 3 |
| 8100 | 55.38 | 0.35 | 3 |
| 8963 | 31.39 | 0.41 | 4 |
| 8970 | 39.86 | 0.48 | 2 |
| 8974 | 39.76 | 0.51 | 3 |
| 8976 | 42.60 | 0.52 | 3 |
| 9071 | 62.17 | 0.42 | 4 |
| 9214 | 27.29 | 0.50 | 2 |
| 9292 | 35.42 | 0.44 | 9 |
| 9497 | 51.92 | 0.33 | 4 |
| 9499 | 25.85 | 0.36 | 4 |
| 9500 | 38.16 | 0.35 | 3 |
| 9538 | 62.20 | 0.38 | 8 |
| 9607 | 36.12 | 0.51 | 4 |
| 9608 | 41.56 | 0.36 | 8 |
| 9697 | 19.70 | 0.30 | 4 |
| 9700 | 55.50 | 0.47 | 3 |
| 9701 | 44.31 | 0.36 | 10 |
| 9720 | 54.75 | 0.43 | 6 |
| 9721 | 34.05 | 0.53 | 3 |
| 23148 | 46.59 | 0.32 | 4 |
| 23315 | 62.61 | 0.42 | 3 |
| 23674 | 38.91 | 0.61 | 3 |
| 24138 | 66.60 | 0.35 | 2 |
| 24140 | 44.08 | 0.41 | 4 |
| 24342 | 22.55 | 0.51 | 4 |
| 24343 | 63.72 | 0.37 | 3 |
| 24344 | 58.36 | 0.50 | 4 |
| 24387 | 48.41 | 0.40 | 4 |
| 24392 | 61.25 | 0.36 | 3 |
| 24393 | 63.22 | 0.47 | 3 |
| 24409 | 46.20 | 0.38 | 4 |
| 24410 | 35.92 | 0.34 | 3 |
| 24414 | 78.36 | 0.40 | 4 |
| 24415 | 42.50 | 0.43 | 3 |
| 24417 | 65.37 | 0.46 | 2 |
| 24909 | 34.99 | 0.43 | 4 |
| 24921 | 43.87 | 0.40 | 4 |
| 24926 | 43.26 | 0.34 | 7 |
| 24927 | 42.20 | 0.42 | 4 |
| 24952 | 46.63 | 0.36 | 4 |
| 24953 | 30.83 | 0.40 | 4 |
| 24955 | 73.66 | 0.42 | 3 |
| 24980 | 89.14 | 0.34 | 3 |
| 24990 | 55.12 | 0.37 | 3 |
| 25005 | 76.10 | 0.32 | 2 |
| 25008 | 34.73 | 0.41 | 9 |
| 25054 | 35.23 | 0.39 | 4 |
| 25077 | 45.73 | 0.39 | 3 |
| 25116 | 65.72 | 0.45 | 7 |
| 25117 | 59.78 | 0.36 | 4 |
| 25123 | 51.08 | 0.50 | 2 |
| 25388 | 36.39 | 0.45 | 4 |
| 25389 | 30.78 | 0.41 | 3 |
| 25413 | 54.01 | 0.48 | 2 |
| 25444 | 38.43 | 0.48 | 3 |
| 25688 | 47.22 | 0.68 | 2 |
| 25694 | 48.82 | 0.42 | 8 |
| 26507 | 38.69 | 0.38 | 4 |
| 26534 | 79.46 | 0.39 | 4 |
| 26538 | 49.53 | 0.52 | 3 |
| 26832 | 71.32 | 0.36 | 3 |
| 26833 | 62.48 | 0.36 | 7 |
| 26839 | 36.26 | 0.37 | 8 |
| 27346 | 33.49 | 0.50 | 4 |
| 27361 | 36.27 | 0.44 | 6 |
| 27362 | 50.01 | 0.38 | 4 |
| 27368 | 46.68 | 0.39 | 10 |
| 27369 | 30.22 | 0.61 | 8 |
| 27375 | 39.71 | 0.40 | 4 |
| 27580 | 53.53 | 0.45 | 4 |
| 27912 | 52.53 | 0.42 | 3 |
| 28827 | 51.47 | 0.53 | 2 |
| 29027 | 47.40 | 0.43 | 4 |

*Every deficiency is recorded in the presence of the heterozygous *rim*^103^ allele.

**Deficiencies are identified by the stock number according to Bloomington Drosophila Stock Center.

***All genotypes were rebalanced to *Tm6B/Tb* balancer prior to performing the screen.

**Table S3 (referring to Figure 2)**

Values inclusive of additional validation for hits obtained in the forward genetic screen.

| **Hits from the screen** | | | | | |
| --- | --- | --- | --- | --- | --- |
| **deficiency ID** | **mEPSP (mean ± sem)** | | **quantal content (mean ± sem)** | | **n** |
| 7562 | 0.42 | ±0.01 | 30.54 | ±1.64 | 22 |
| 7578 | 0.47 | ±0.02 | 32.36 | ±2.52 | 16 |
| 7596 | 0.34 | ±0.04 | 35.70 | ±6.79 | 12 |
| 7657 | 0.45 | ±0.02 | 37.78 | ±3.09 | 11 |
| 7921 | 0.34 | ±0.02 | 36.67 | ±3.26 | 10 |
| 7963 | 0.35 | ±0.01 | 32.01 | ±2.44 | 8 |
| 7990 | 0.38 | ±0.06 | 33.10 | ±3.73 | 4 |
| 7992 | 0.41 | ±0.02 | 28.17 | ±2.41 | 13 |
| 7997 | 0.39 | ±0.02 | 27.63 | ±4.12 | 6 |
| 8097 | 0.40 | ±0.04 | 26.16 | ±8.67 | 3 |
| 8963 | 0.43 | ±0.03 | 31.12 | ±3.44 | 6 |
| 9214 | 0.44 | ±0.03 | 35.41 | ±3.83 | 6 |
| 9499 | 0.44 | ±0.03 | 27.45 | ±2.47 | 12 |
| 9697 | 0.32 | ±0.01 | 25.12 | ±4.12 | 7 |
| 24342 | 0.45 | ±0.03 | 26.05 | ±2.02 | 7 |
| 24410 | 0.34 | ±0.01 | 41.14 | ±4.43 | 13 |
| 24909 | 0.43 | ±0.02 | 34.99 | ±5.36 | 4 |
| 24953 | 0.40 | ±0.02 | 28.67 | ±2.32 | 7 |
| 25389 | 0.46 | ±0.02 | 24.39 | ±2.69 | 7 |

*Every deficiency is recorded in the presence of a heterozygous *rim*^103^ allele.

**Deficiencies are identified by the stock number according to Bloomington Drosophila Stock Center.

***All genotypes are rebalanced to *Tm6B/Tb* balancer prior to the screen.

**Table S4 (referring to Figure 2).**

Genotypic information for hits selected in the forward genetic screen.

| **deficiency ID** | **Original stock** | **Chromosomal location** | **Break points** |
| --- | --- | --- | --- |
| 7562 | w1118; Df(3L)Exel6083, P{XP-U}Exel6083/TM6B, Tb1 | 3L:104350;3L:180193 (Df) | 61A6;61B2 (Df) |
| 7578 | w1118; Df(3L)Exel6099, P{XP-U}Exel6099/TM6B, Tb1 | 3L:3925483;3L:4073993 (Df) | 63F7;64A5 (Df) |
| 7596 | w1118; Df(3L)Exel6117, P{XP-U}Exel6117/TM6B, Tb1 | 3L:12627834;3L:12789933 (Df) | 69D1;69E2 (Df) |
| 7657 | w1118; Df(3R)Exel6178, P{XP-U}Exel6178/TM6B, Tb1 | 3R:18166427;3R:18397356 (Df) | 90F4;91A5 (Df) |
| 7921 | w1118; Df(3L)Exel9000/TM6B, Tb1 | 3L:4227243;3L:4284262 | 64A10;64A12 (Df) |
| 7963 | w1118; Df(3R)Exel8153/TM6B, Tb1 | 3R:11435909;3R:11569171 (Df) | 86E5;86E11 (Df) |
| 7990 | w1118; Df(3R)Exel9012/TM6B, Tb1 | 3R:23279758;3R:23346387 (Df) | 94E9;94E13 (Df) |
| 7992 | w1118; Df(3R)Exel9014/TM6B, Tb1 | 3R:23773121;3R:23943004 (Df) | 95B1;95D1 (Df) |
| 7997 | w1118; Df(3R)Exel7378/TM6B, Tb1 | 3R:30563224;3R:30794955 (Df) | 99F8;100A5 (Df) |
| 8097 | w1118; Df(3L)ED4502, P{3'.RS5+3.3'}ED4502/TM6C, cu1 Sb1 | 3L:13227765;3L:13993551 (Df) | 70A3;70C10 (Df) |
| 8963 | w1118; Df(3R)ED6103, P{3'.RS5+3.3'}ED6103/TM6C, cu1 Sb1 | 3R:22898553;3R:23258415 (Df) | 94D3;94E9 (Df) |
| 9214 | w1118; Df(3L)ED4536, P{3'.RS5+3.3'}ED4536/TM6C, cu1 Sb1 | 3L:14002761;3L:14205324 (Df) | 70C11;70D3 (Df) |
| 9499 | w1118; Df(3R)BSC139/TM6B, Tb+ | 3R:5264002;3R:5445840 (Df) | 82F8;83A3 (Df) |
| 9697 | w1118; Df(3L)BSC220/TM6C, Sb1 cu1 | 3L:18972562-18972825;3L:19171268 (Df) | 75F1;76A1 (Df) |
| 24342 | w1118; Df(3R)BSC316/TM6B, Tb+ | 3R:4387566-4387611;3R:4453290 (Df) | 82A5;82B2 (Df) |
| 24410 | w1118; Df(3L)BSC386/TM6C, Sb1 cu1 | 3L:3047163;3L:3169058 (Df) | 63A3;63B8 (Df) |
| 24909 | w1118; Df(3R)BSC321/TM6C, Sb1 cu1 | 3R:25637827;3R:25680519 (Df) | 96E6;96E9 (Df) |
| 24953 | w1118; Df(3L)BSC449/TM6C, Sb1 cu1 | 3L:20856915-20856988;3L:21202930 (Df) | 77F2;78C2 (Df) |
| 25389 | w1118; Df(3R)BSC566/TM6C, Sb1 | 3R:17755304;3R:18198213 (Df) | 90C2;90F6 (Df) |
| 27346 | w1118; Df(3L)BSC774/TM6C, Sb1 cu1 | 3L:15699903;3L:16240273-16240280 (Df) | 71F1;72D10 (Df) |

**Table S5 (referring to Figure 2)**

Genes uncovered within the deficiencies identified as hits in the forward genetic screen.

| **Deficiency ID** | **Genes uncovered by each deficiency hits** |
| --- | --- |
| 7562 | pdk1, CG6845, Dic61B, p130CAS, CG7049, Vdup1 |
| 7578 | CG10866, CG12766, CG10863, CG14982, CG10853, CG14983, CG14984, CG12605, wit, dib, cg1136, cg14985, foxl1, Mul1,cg11594, Gr64a, Gr64c, Gr64b, Gr64f, cg11593 |
| 7596 | MIRR, SmD1, Ptp69D, CG32112, CG32109, Klc, CG10973, Hip1, CG32106, CG10969, CG17166, Atg1 |
| 7657 | Cdm, CG12320, CG12321, CG14309, CG14312, CG14313, CG14314, CG15803, CG18598, CG18599, CG18600, CG44158, CG7126, CG7131, CG7142, CG7156, CG7168, CG7183, CG7208, CG7215, CG7675, CG7985, CG7988, CG8064, Cona, Dlc90F, eIF1A, gl hmw Mdh2, MED17, Moi, mTerf5, naz, Non3, Nup43, PKD, Prx5, Repo, Rpb4, Ssdp, Tgs1, Vha100-2, Vha100-4, Vps39 Vti1b, Wrd, WRNexo |
| 7921 | Ago, CG1265, CG1273, CG1309, CG11583, CG11586, CG14997, CG15011, Ctl1, Pav, srw, VhaM9.7-a |
| 7963 | CG14708, CG17230, DPR4, DPR5, PGRP-LB, SALS, CG10898, SEA,FABP |
| 7990 | ORB, CDC16, CG6763, CG17083, CG4434, PNT |
| 7992 | MBC, CG33111, CG34355, EIF4G2, PLI, tbrd-1, tfIIa-s, sba, ndc1, gdh, cg13601, cg43998, cg43999, cg31142, rpt2, cg13599, rps19b, cg5854 |
| 7997 | TMOD, CG9702, RPT6R, CG9717, CG2246, CG31019, CG3102, PH4ALPHANE3, CG31016, CG44954, CG2267, CG31013, PH4alphaPV, SPN100A, CG12069, PKA-C2, CG31010, CG1340, CG11313, CG15543, NPC2G, NPC2H, ZFH1, WTS |
| 8097 | CAPS, CG17687, Nplp2, CG14111, SNCF, CG14107, ImpL1, CG14110, CG10171, Poc1, Sens, CG10222, Flr, CG32121, CG33263, CG14106, CG14105, CG10713, CG10154, CG10725, CG10140, CG14109, CG10732, CG10133, CG10738, CG10116, CG10089, Stv, Abp1, Tgi, Spt20, Vps36, Liprin-beta, CG10710, bru-3, CG43184, CG8757, CG8750, Tsp68C, Hml, CG8745, Dysc, CG13737, Rgl, CG8833, DCTN1-p150, CG32137, Meics, Nxf3, ssp2, Hsc70-1, CG17364 |
| 8963 | CenB1A, CG13827, CG13829, CG13833, CG13837, CG1383, CG17109, CG17110, CG17111, CG17119, CG17121, CG31365, CG31457, CG34375, CG4467, CG46310, CG6660, CG6688, CG6726, CG6733, CG6738, cnc, Cow, EloA, fzo, Gbp3, Gr94a, hh, Irk1, klg, lmd, Nha2, Or94a, Or94b, p53, Rad60, Rassf, sav, Tpl94D, Ublcp1, unk, VhaAC39-2, wda |
| 9214 | CG6833, Hsc70Cb, Ptip, CG8100, Saturn, btlD, nanSox21a, CG13484, Endos, Neurl4, Sox21b, CG6650, Fbp1, Nuf, upset, CG6661, Frl, Pex1 |
| 9499 | Mtd, CG31538, Tim17b1, CG11999, CG1161, Prosbeta7, Cerk, CG34277, CG31542, RpII18, Hd, CG14667, 7B2, Kkv, CG14668, CG1172, Or83a, CG2663, Orco, CG14669 |
| 9697 | Acp76A, CG11619, CG14075, CG14079, CG14082, CG18135, CG34256, CG3808, CG43407, CNPYb, fz2, Gem2, MESR6, Mkp3, Naxd, nkd, Sfxn2, SmydA-2, Spn75F, Ugt316A1 |
| 24342 | CG1074, CG14646, CG14647, CG14650, CG31522, CG31525, CG31528 CG32944, CG34112, CG9804, CG9853, CG9855, eIF3a, lost, srl, tub |
| 24410 | SK2, CG32485, CG1271, CG16753, CG32486, CHT7, PAN3, PROMININ-LIKE, CG45066, CG45067, CG11537, CG1291, CG12082, BTBVII, CG15812, ASCIZ, CG32280, CG32281 |
| 24909 | jigr1, Tnks, RASSF8, Lgr3, CG4730, CG5039, CG4743 |
| 24953 | Ac78C, AsnS, asRNA:CR45801, CG10512, CG10565, CG10581, CG10584, CG10587, CG10588, CG10589 CG11037, CG11458, CG12983, CG12984, CG32432, CG33054, CG33056, CG33284, CG33285, CG33286 CG33287, CG34261, CG42337, CG43072, CG43931, CG43938, chb, fng, ICA69, ko, park, Pdss2, Sems, Sfp77F, Sin, siz, skd |
| 25389 | TyrR, CG43102, CG7713, CG7379, CG17803, CG17806, CG17802, CG17801, CG7357, CG18012, Tinc, Rim, CG12347, CG43445, Cpo, DNaseII, CG7785, CG7794, Htl, CG14317, Sr, CG14316, CG14315, CG7218, Cbp20, Prx5, CG7215, wrd, Cdm, CG7208, Arp5, CG12321, MED17, CG14313, Ssdp |
| 27346 | CG42570, Comm, CG6244, CG13445, Few, CkIIalpha-i1, DCP2, Dbo, CG12713, CG18081, CG15715, CG32150, pHCl, sff, Pka-C3, GXIVsPLA2, Elgi, CG17032, l(3)72Ab, CG10516, Brm, CG17026, CG17029, CG17028, CG17027, Arl1, DNApol-delta, Arl1, DNApol-delta, mRpS31, mib1, Notum, CG42717, CG42716, CG42538, CG5895, Diap1, Mbs, CG33258, CG13075, Taspase1, CG5235, ClC-c, CG13074, ms(3)72Dt, CG5414, IntS9, CG43295, Zn72D, Taf4, CG12272, Pgm, SsRbeta, Trs20, elg1, CG5157, CG5151, CG32152, CG33796, CG33795, CG33687, CG33688, CG33689, CG33690, CG13073, l(3)72Dn, CG5027, PDCD-5, MED10, Hsc20, l(3)72Dp |

**Table S6 (referring to Figures 1 and 4)**

Average values for data presented in Figure 1 and Figure 4

| **Genotype** | **mEPSP**  **(-/+PhTx)** | **P-value** | **EPSP**  **(-/+PhTx)** | **P-Value** | **Quantal Content  (-/+PhTx)** | **P-value** | **n (-PhTx)** | **n (+ PhTx)** |
| --- | --- | --- | --- | --- | --- | --- | --- | --- |
| w1118/+ | 0.77/0.40 | 4.07E-19 | 33.3 / 29.7 | 0.00275 | 44.1 / 77.1 | 4.38E-17 | 47 | 36 |
| 7562/+ | 0.76/0.41 | 2.21E-06 | 34.1 / 22.1 | 0.00114 | 44.8/ 57.5 | 0.09556 | 6 | 7 |
| 8963/+ | 0.81/0.37 | 4.26E-08 | 35.7 / 21.8 | 0.00000031 | 44.2 / 58.9 | 0.00064 | 8 | 10 |
| 7963/+ | 0.84/0.34 | 0.000000000136 | 28.8 / 23.4 | 0.31996 | 35.2/ 68.3 | 0.00301 | 8 | 8 |
| 24410/+ | 0.81/0.34 | 0.000000163 | 31.3 / 21.8 | 0.00434 | 39.5 / 65.3 | 0.00029 | 8 | 11 |
| 24953/+ | 0.80/0.41 | 4.71E-14 | 26.1/ 22.9 | 0.19476 | 33.2/ 56.1 | 0.000061 | 13 | 15 |
| CHD2/+ | 0.85/0.40 | 0.000104 | 28.7/ 25.6 | 0.27 | 36.2 / 64.3 | 0.00003 | 8 | 19 |
| CHD2/+;7562/+ | 0.69/0.37 | 0.000000102 | 30.7 / 14.2 | 0.000017 | 44.6/ 38.0 | 0.19615 | 4 | 10 |
| CHD2/+;8963/+ | 0.69/0.37 | 0.00015 | 26.8/ 17.4 | 0.00358 | 38.6 / 47.0 | 0.00919 | 7 | 11 |
| CHD2/+;24410/+ | 0.96/0.44 | 0.00001 | 31.9 / 19.1 | 0.0098 | 32.8/ 45.1 | 0.122 | 8 | 7 |
| CHD2/+;24953/+ | 0.93/0.48 | 0.00121 | 21.1 / 12.7 | 0.00282 | 23.9 / 26.3 | 0.56128 | 8 | 12 |
| CHD8/+ | 0.61/0.41 | 0.00461 | 29.9 / 25.8 | 0.09814 | 51.3 / 66.3 | 0.04464 | 7 | 8 |
| CHD8/+;7562/+ | 0.48/0.45 | 0.50201 | 23.8 / 21.6 | 0.3212 | 52.1 / 48.8 | 0.6747 | 8 | 10 |
| CHD8/+;8963/+ | 0.78/0.46 | 0.00061 | 29.9 / 20.9 | 0.00306 | 38.8 / 46.6 | 0.02838 | 9 | 17 |
| CHD8/+;7963/+ | 0.63/0.35 | 0.00000223 | 23.8 / 18.7 | 0.00843 | 41.1 / 53.7 | 0.06365 | 8 | 11 |
| CHD8/+;24410/+ | 0.65/0.33 | 0.00041 | 24.9 / 17.0 | 0.000087 | 39.0 / 44.4 | 0.21624 | 6 | 14 |
| CHD8/+;24953/+ | 0.60/0.41 | 0.00000157 | 21.5 / 16.6 | 0.01199 | 36.8 / 41.2 | 0.26813 | 16 | 16 |
| WDFY3/+ | 0.58/0.37 | 0.00103 | 29.7 / 24.9 | 0.29837 | 51.4/ 68.0 | 0.05123 | 8 | 7 |
| WDFY3/+;7562/+ | 0.67/0.32 | 0.00269 | 25.1 / 14.3 | 0.00314 | 40.4 / 44.6 | 0.51348 | 7 | 8 |
| WDFY3/+;8963/+ | 0.73/0.43 | 0.000000405 | 30.1 / 24.3 | 0.13024 | 33.3 / 54.2 | 0.00977 | 7 | 7 |
| WDFY3/+;7963/+ | 0.60/0.47 | 0.00126 | 27.5 / 27.6 | 0.982 | 45.9 / 59.3 | 0.0209 | 8 | 11 |
| WDFY3/+;24410/+ | 0.61/0.40 | 0.00138 | 24.1 / 19.7 | 0.09596 | 40.1 / 49.6 | 0.10056 | 8 | 8 |
| WDFY3/+;24953/+ | 0.64/0.38 | 0.0000106 | 22.9 / 10.9 | 0.00472 | 35.6 / 28.5 | 0.21712 | 8 | 9 |
| RIMS1/+ | 0.73/0.40 | 1.15E-11 | 27.5 / 25.1 | 0.02848 | 38.5 / 64.4 | 0.0000000051 | 20 | 30 |
| RIMS1/+,7562/+ | 0.67/0.40 | 0.00092 | 18.6 / 12.6 | 0.00521 | 27.9 / 31.3 | 0.16438 | 7 | 18 |
| RIMS1/+,8963/+ | 0.73/0.43 | 0.01022 | 16.0 / 13.3 | 0.26477 | 22.6 / 31.1 | 0.05777 | 6 | 9 |
| RIMS1/+,7963/+ | 0.60/0.35 | 0.00782 | 25.9 / 11.4 | 0.00105 | 43.1 / 32.0 | 0.00384 | 4 | 8 |
| RIMS1/+,24410/+ | 0.63/0.34 | 0.000000254 | 20.6 / 13.3 | 0.00619 | 33.1 / 41.1 | 0.3635 | 7 | 13 |
| RIMS1/+,24953/+ | 0.61/0.40 | 0.0000232 | 19.4 / 12.3 | 0.0025 | 31.8 / 28.5 | 0.32924 | 6 | 7 |

| **Genotype** | **mEPSP**  **(-/+PhTx)** | **P-value %change**  **(-/+ PhTx)** | **Quantal Content (-/+PhTx)** | **P-value**  **%change**  **(-/+ PhTx)** | **n**  **(-PhTx)** | **n**  **(+ PhTx)** |
| --- | --- | --- | --- | --- | --- | --- |
| PPP2R5D/+;  w1118/+ | 0.92 / 0.52 | 1.325E-11 | 33.02 / 57.02 | 1.38E-06 | 8 | 16 |
| PPP2R5D/+;  RIMS1/+ | 0.74 / 0.41 | 1.1E-07 | 30.55 / 36.59 | 0.026 | 20 | 25 |
| PPP2R5D/+;  CHD8/+ | 0.65 / 0.35 | 1.94E-08 | 42.26 / 44.56 | 0.675 | 11 | 14 |
| PPP2R5D/+;  CHD2/+ | 0.85 / 0.44 | 0.056 | 44.37 / 52.74 | 0.202 | 4 | 7 |
| PPP2R5D/+;  WDFY3/+ | 0.75 / 0.47 | 4.23E-07 | 39.17 / 35.78 | 0.589 | 4 | 10 |
| PPP2R5D/+;  ASH1L/+ | 0.67 / 0.43 | 0.001 | 35.94 / 53.07 | 0.001 | 6 | 12 |
| PDPK1/+;  w1118/+ | 0.70 / 0.43 | 6.13E-07 | 37.84 / 66.34 | 0.0009 | 4 | 8 |
| PDPK1/+;  RIMS1/+ | 0.78 / 0.42 | 0.0003 | 36.80 / 33.15 | 0.442 | 10 | 25 |
| PDPK1/+;  CHD8/+ | 0.71 / 0.43 | 0.0901 | 35.58 / 47.38 | 0.055 | 6 | 8 |
| PDPK1/+;  CHD2/+ | 0.73 / 0.45 | 2.9E-07 | 42.25 / 48.55 | 0.059 | 4 | 8 |
| PDPK1/+;  WDFY3/+ | 0.75 / 0.47 | 0.0003 | 38.96 / 42.25 | 0.0018 | 11 | 18 |
| PDPK1/+;  ASH1L/+ | 0.88 / 0.54 | 0.0004 | 28.05 / 45.03 | 0.277 | 12 | 9 |

**Table S7 (referring to Figure 5)**

Average values for the data presented in Figure 5

*See legend, Figure 5, for details regarding the statistical tests used to generate stated P-value
